# What evidence exists on the links between natural climate solutions and climate change mitigation outcomes in subtropical and tropical terrestrial regions? A systematic map protocol

**DOI:** 10.1186/s13750-022-00268-w

**Published:** 2022-04-19

**Authors:** Samantha H. Cheng, Sebastien Costedoat, Eleanor J. Sterling, Catherine Chamberlain, Arundhati Jagadish, Peter Lichtenthal, A. Justin Nowakowski, Auset Taylor, Jen Tinsman, Steven W. J. Canty, Margaret B. Holland, Kelly W. Jones, Morena Mills, David Morales-Hidalgo, Starry Sprenkle-Hyppolite, Meredith Wiggins, Michael B. Mascia, Carlos L. Muñoz Brenes

**Affiliations:** 1 Center for Biodiversity and Conservation, American Museum of Natural History, New York, NY USA; 2 Moore Center for Science, Conservation International, Arlington, VA USA; 3 Columbia University, New York, NY USA; 4 Working Land and Seascapes, Conservation Commons, Smithsonian Institution, Washington, DC USA; 5 Smithsonian Marine Station, Fort Pierce, FL USA; 6 Department of Geography & Environmental Systems, University of Maryland Baltimore County, Baltimore, MD USA; 7 Human Dimensions of Natural Resources Department, Colorado State University, Fort Collins, CO USA; 8 Imperial College London, London, UK; 9 Forestry Division, Food and Agriculture Organization of the United Nations, Rome, Italy; 10 Center for Natural Climate Solutions, Conservation International, Arlington, VA USA; 11 DAI, Washington, DC USA

**Keywords:** Natural climate solutions, Climate change, Nature-based solutions, Mitigation, Land cover and land use change, Conservation, Restoration, Land management

## Abstract

**Background:**

Natural climate solutions (NCS)—actions to conserve, restore, and modify natural and modified ecosystems to increase carbon storage or avoid greenhouse gas (GHG) emissions—are increasingly regarded as important pathways for climate change mitigation, while contributing to our global conservation efforts, overall planetary resilience, and sustainable development goals. Recently, projections posit that terrestrial-based NCS can potentially capture or avoid the emission of at least 11 Gt (gigatons) of carbon dioxide equivalent a year, or roughly encompassing one third of the emissions reductions needed to meet the Paris Climate Agreement goals by 2030. NCS interventions also purport to provide co-benefits such as improved productivity and livelihoods from sustainable natural resource management, protection of locally and culturally important natural areas, and downstream climate adaptation benefits. Attention on implementing NCS to address climate change across global and national agendas has grown—however, clear understanding of which types of NCS interventions have undergone substantial study versus those that require additional evidence is still lacking. This study aims to conduct a systematic map to collate and describe the current state, distribution, and methods used for evidence on the links between NCS interventions and climate change mitigation outcomes within tropical and sub-tropical terrestrial ecosystems. Results of this study can be used to inform program and policy design and highlight critical knowledge gaps where future evaluation, research, and syntheses are needed.

**Methods:**

To develop this systematic map, we will search two bibliographic databases (including 11 indices) and 67 organization websites, backward citation chase from 39 existing evidence syntheses, and solicit information from key informants. All searches will be conducted in English and encompass subtropical and tropical terrestrial ecosystems (forests, grasslands, mangroves, agricultural areas). Search results will be screened at title and abstract, and full text levels, recording both the number of excluded articles and reasons for exclusion. Key meta-data from included articles will be coded and reported in a narrative review that will summarize trends in the evidence base, assess gaps in knowledge, and provide insights for policy, practice, and research. The data from this systematic map will be made open access.

**Supplementary Information:**

The online version contains supplementary material available at 10.1186/s13750-022-00268-w.

## Background

Addressing the drivers of climate change and mitigating the impacts humanity already confronts is at the top of global priorities. There is widespread concern and increased ambition to prevent global warming from reaching or exceeding 2 °C and meet the Paris Climate Agreement goals. Amongst these calls for action, there is a major focus on improving stewardship of terrestrial ecosystems thereby strengthening the role these systems can play in achieving climate change mitigation outcomes. Those activities that fall within the agricultural, forestry, and other land use (AFOLU) sector are estimated to contribute about one-fifth of global greenhouse gas (GHG) net emissions through degradation and conversion of natural ecosystems (e.g. forest and grasslands) and agricultural production [1]. Simultaneously, these ecosystems play a crucial role in mitigating climate change as they remove carbon dioxide emissions from the atmosphere—by storing and sequestering carbon above and below ground [1, 2].

In the wake of the global COVID-19 pandemic and increasing incidences of climate change-related floods, extreme heat, and storms [3]—global agendas have wholly pivoted to addressing climate change (e.g. [4–6]. The unpredictability and severity of these rapid-onset shocks erode the already waning resilience of communities and nations to manage the impacts of the ongoing degradation of critical ecosystems and resources. Additionally, they impede the ability to plan and respond to the longer-term slow-onset effects of the climate crisis like drought or sea-level rise. Over the past two decades, international policy initiatives such as Reducing Emissions from Deforestation and Forest Degradation and the role of conservation, sustainable management of forests, and enhancement of forest carbon stocks in developing countries (REDD +), and advances in scientific research and new technologies have propelled several types of nature-based approaches to address climate change and sustainable development. While initiatives such as protection of natural ecosystems, sustainable management of terrestrial resources (e.g. forests, grasslands, and peatlands), and improved agricultural practices (e.g. conservation agriculture and optimizing grazing) are not new—efforts to explicitly improve and implement these practices for climate change mitigation are increasing.

‘Natural Climate Solutions’ (NCS) have recently gained attention as a set of highly promising pathways for climate change mitigation through activities taking place in natural and modified ecosystems. NCS falls within the broader scope of “nature-based solutions” (NbS) and ecosystem-related approaches which aim to protect, sustainably manage, and restore natural and modified ecosystems to address societal challenges as a whole—including efforts to improve resiliency and adaptive capacity to climate change [7]. NbS emphasizes implementation that supports and prioritizes nature and people [8]. NCS are comprised of more narrow subset of ecosystem stewardship activities (such as protecting, restoring, and/or managing forests, mangroves, peatlands, grasslands, and agricultural ecosystems) that have the potential to mitigate climate change [2, 9]. This includes both reductions in GHG emissions (NO_2_, CH_4_, CO_2_) in all economic sectors, and increased carbon storage and sequestration of above- and below-ground carbon. Within the larger global efforts needed to achieve climate change mitigation, NCS could represent at least a third of the total cost-effective mitigation potential, with the highest potential in tropical countries [2, 9]. Indeed, land-based interventions could mitigate up to 8–13.8 GtCO_2_eq yr^−1^ between 2020 and 2050 at less than US$ 100/tCO_2_eq [10]. In addition, NCS are generally regarded as “low-tech” solutions and may present a feasible, scalable, and cost-effective strategy for large-scale climate change mitigation. Given these features, there is significant attention on whether NCS may be a useful approach, particularly for countries with limited resources [9, 10]. NCS can also induce important co-benefits for biodiversity and other socio-economic outcomes (e.g. [11, 12]).

Addressing climate change will also require clear and explicit consideration of impacts on human society and natural ecosystems. In general, the impacts of climate change are felt most acutely by individuals, communities, and countries who contribute the least to global emissions and are often the most vulnerable due to their location or history of colonialist rule and exploitation. Thus, NCS that are also targeted at increasing the resilience and adaptive capacities of ecosystems and communities are key to mitigating the ongoing and future impacts of climate change. NCS may provide important co-benefits in the form of adaptation, biodiversity maintenance, and other essential provisioning, regulating and cultural ecosystem services, which are fundamental for achieving the Sustainable Development Goals alongside the goals of the Paris Climate Agreement [9, 13]. For example, climate-smart agricultural practices aim to reduce GHG emissions while also enhancing the adaptive capacity of farmers to cope with the ongoing impacts of climate change [14, 15]. However, NCS, like other types of conservation and natural resource management interventions can have unintended and negative consequences for local communities and ecosystems [16, 17]. For example, some area protection and restoration activities have ignored practices of free, prior, and informed consent and disregarded customary rights of Indigenous Peoples and Local Communities (IPLCs) which has resulted in inequitable and potentially detrimental impacts on human well-being, and ultimately on ecosystems.

To slow down or halt climate change impacts and improve and maintain the well-being of nature and people, we need to understand the ability of different types of actions to achieve climate change mitigation outcomes. Nevertheless, the extent of evidence about how much change in land-based mitigation outcomes (e.g. additionally avoided CO_2_-equivalent emissions or enhanced carbon sequestration, or their respective land use/land cover proxies) can be attributed to specific interventions (i.e. policies, programs, and projects) is often not rigorously evaluated. Moreover, estimates of potential sequestration of different land-use and interventions from stand-alone modeling or estimation studies have met considerable debate (e.g. [18, 19]). There have been several systematic studies and meta-analyses characterizing the evidence base about the link between various types of NCS interventions and climate mitigation outcomes. An evidence gap map from the International Initiative for Impact Evaluation (3ie) on the impact of land use change and forestry programs on GHG emissions and food security has helped illuminate the state of evidence in this topic, taking into account various conservation interventions (e.g. protected areas, community-based conservation, payments for environmental services) [20]. A recent update of this evidence gap map showed that the evaluation of forest conservation outcomes has considerably grown in recent years, but that the evidence base remains insufficient across most forest conservation intervention types, and notably as it relates to climate mitigation outcomes [21]. Several global meta-analyses comparing the effectiveness of various approaches to ecosystem restoration (e.g. passive restoration, assisted natural regeneration, and active plantation), and other approaches following the principles of Forest Landscape Restoration (FLR)) also highlighted that evidence exists that some interventions have been effective in enhancing carbon sequestration, but require attention to improving the evidence base, notably by more rigorously disentangling the net impact of specific restoration interventions from confounding factors and site location bias (see e.g. [22–24]). An evidence gap map looking specifically at the environmental impacts of agroforestry also found that most studies employ study designs that are not well-suited for broader causal inference [25]. As well, a multi-sites pantropical study measured the climate mitigations outcomes resulting from reduced-impact logging in several projects located in 7 tropical countries [26]. Recently, a systematic map highlighted that the evidence base for climate-smart agriculture in East and Southern Africa was skewed towards just a few commodities and geographies [27].

While this growth in syntheses is encouraging, most efforts have been limited in scope—for example, focusing on just one or two action types or NCS pathways or in single countries or regions. Thus, there is a need for a comprehensive characterization of the evidence base covering interventions relevant for the full range of NCS pathways and their links with land-use and land-cover change (LULCC), and mitigation outcomes to inform future planning and prioritization for action and evaluation. This study aims to collate and summarize the existing evidence on the impacts of all classes of NCS interventions on climate change mitigation outcomes in tropical countries, to characterize the evidence base and gaps, and to communicate data and products to key decision makers and researchers in relevant sectors and organizations. A comprehensive synthesis is timely to determine where the current state of knowledge is versus where we need to direct future research and make progress addressing climate change through interventions within natural ecosystems.

## Stakeholder engagement

The formulation of this research question and scope of this systematic map was commissioned by the Moore Center for Science at Conservation International (CI) to accelerate learning, inform the design, and scaling of NCS. Thus, this systematic map will advance understanding of the current extent of how impact is being evaluated within the NCS evidence base, to better prioritize areas for future research and investment for both CI as well as organizations, agencies, and other institutional actors addressing climate change. The project convened and engaged with a *stakeholder advisory group* of researchers, practitioners, and policymakers who work in this sector, to provide input into the scope and interpretation of the insights from this map. The *advisory group* provided key input into the elements of the synthesis questions, shaped the framework of this synthesis (see “Framework development” section), and provided suggestions for relevant literature and online sources of information (particularly grey literature). The *evidence synthesis team* is composed of evidence synthesis scientists, topic area experts, and practitioners and leads the development and execution of this study. The *synthesis* team is led by the Center for Biodiversity and Conservation at the American Museum of Natural History, which is an affiliated center with the Collaboration for Environmental Evidence and focuses on evidence synthesis and evidence-informed decision-making in the conservation and development sectors.

## Objective of this systematic map

The objective of this systematic map is to identify, map, and describe the evidence base surrounding the impacts of NCS interventions on climate change mitigation outcomes (and/or related LULCC impacts) in tropical and subtropical terrestrial ecosystems.

## Question

In this study, we will address the following primary research question(s):

*What is the evidence base for links between NCS interventions and climate change mitigation outcomes in tropical and subtropical forests, grasslands, and agricultural systems?*

*What are the extent and distribution of reviews and meta-analyses that examine links between NCS interventions and climate change mitigation outcomes, intermediate outcomes, biodiversity/ecosystem, or human well-being outcomes in tropical and subtropical forests, grasslands, and agricultural systems?*

We consider direct outcomes for climate change mitigation (e.g. changes to GHG emissions, avoided emissions, carbon storage, carbon sequestration) as well as measures of intermediate environmental change (e.g. changes to forest and land cover, avoided land conversion, land use change – also considered “proxy” measures) directly related to mitigation outcomes. Once we have collated the evidence base, we aim to address the following sub-questions to better characterize the state of evidence to inform future work in NCS design, implementation, and evaluation across objectives for climate change mitigation and co-impacts for nature and people; see “Framework development” section below.

## Sub-questions

### Using the resulting evidence base, we aim to answer the following set of secondary research questions:

- What are the extent and distribution of articles that examine climate change mitigation outcomes while *also* examining co-impacts on intermediate outcomes, biodiversity/ecosystems, and/or human well-being outcomes?
- What are the extent and distribution of articles that examine impacts on belowground mitigation outcomes *in addition to* aboveground impacts?
- What are the extent and distribution of articles that examine different mechanisms through which NCS operate? (e.g. governance, monetary and non-monetary incentives, capacity development, policies and regulations)?
- How are climate mitigation outcomes being measured?
- What study designs are being used to assess the impacts of NCS interventions?

Lastly, we will compare the distribution of studies focused on the co-impacts of NCS interventions and any of the climate change mitigation, biodiversity/ecosystem, and/or human well-being outcomes. This is intended to help understand and characterize the nature of existing knowledge gaps on co-impacts (see Framework Development section below).

## Elements of the primary question

**Table:** 

*Population*	Tropical and subtropical forests, grasslands, mangroves, and agricultural areas (Tropical and Subtropical Coniferous Forests; Tropical and Subtropical Dry Broadleaf Forests; Tropical and Subtropical Grasslands, Savannas and Shrublands; Tropical and Subtropical Moist Broadleaf Forests; Mangroves)
*Intervention*	“Natural Climate Solutions” interventions that aim to protect, manage, and/or restore existing or created ecosystems, as well as those that manage agricultural, forestry, and other land use activities (see Table 2)
*Comparator*	Presence/absence of intervention, temporal (before/after, continuous time series, interrupted time series), spatial (distance), and/or between groups (control/intervention, different interventions, ecosystems, landscapes)
*Outcome*	Climate change mitigation outcomes (in terms of equivalent metric tons of CO_2_) or environmental outcomes directly related to climate change mitigation (e.g. changes to forest and land cover, avoided land conversion, land use change)

### Framework development

**Fig. 1:**
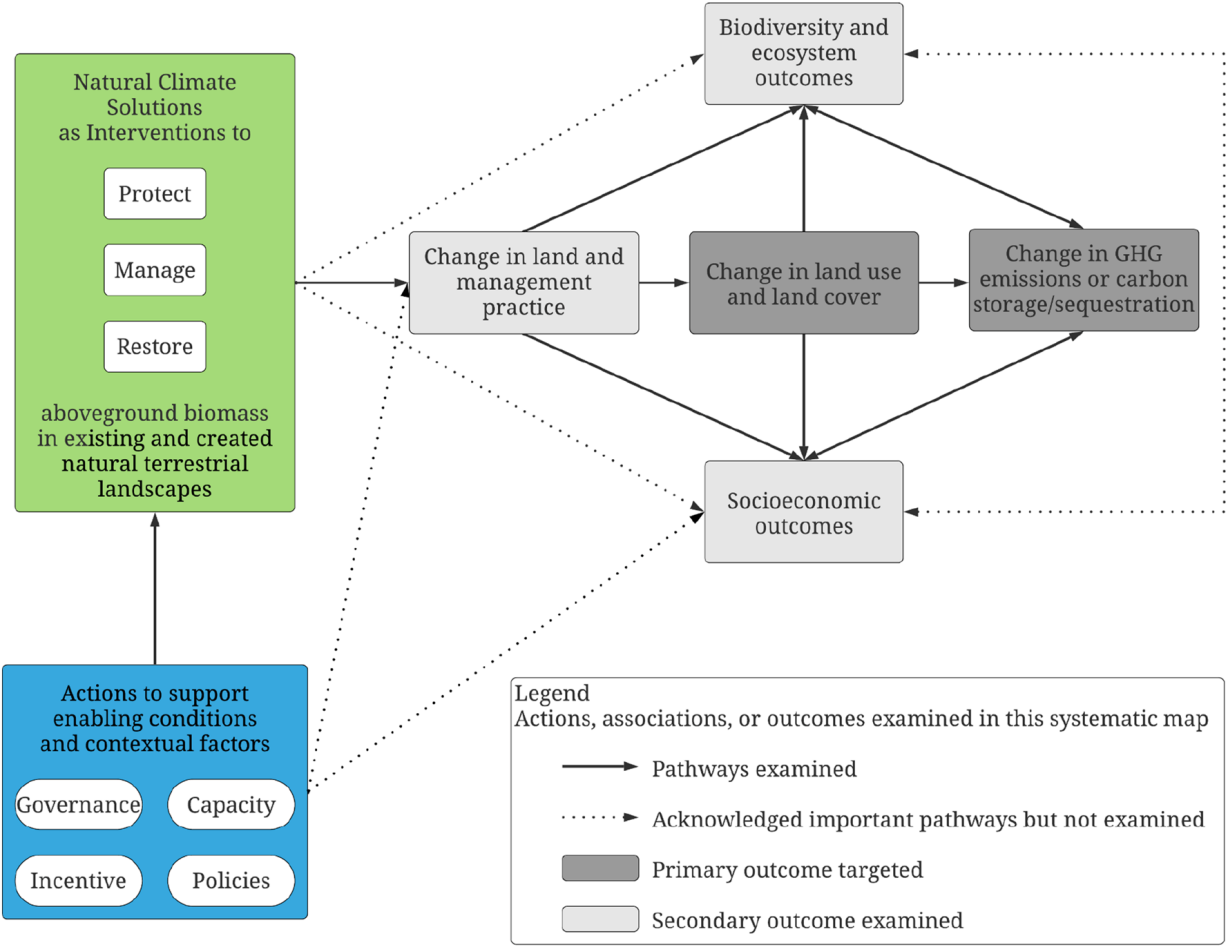
Working theory of change on the links between natural climate solutions (NCS) (green box) and links to change in greenhouse gas emissions and carbon mitigation outcomes (primary outcomes of interest—dark grey). Green box includes the NCS investigated. All aim to increase aboveground biomass in natural and modified terrestrial tropical habitats. They may also have additional, socioeconomic, biodiversity, and ecosystem service outcomes. However, we do not include studies that only focus on these

The framework for this systematic map was developed through a series of discussions and meetings involving the *synthesis team* and *advisory group*. During this process, the overall scope of the project was determined and refined. The framework used in this study reflects a synthesis of existing conceptual models and causal theories on the links between NCS actions and climate change mitigation targets (e.g. [2, 20, 28, 29]) within a unified, generic theory of change (Fig. 1).

NCS interventions (green box in Fig. 1) are often carried out alongside or integrated with actions that aim to support enabling conditions and address contextual factors that influence the effectiveness of NCS interventions (blue box). For example, these include actions to strengthen governance and rights (e.g. formalizing and strengthening land tenure) [30], improving collective action [31], build technical and human capacity (e.g. trainings, capacity development) (e.g. [32]), provide monetary and non-monetary incentives (e.g. REDD + , PES) [33, 34], and enact and enforce policies and regulations and motivate voluntary agreements and partnerships (e.g. Paris Climate Agreement, the Europe Union’s Forest Law Enforcement, Governance and Trade (FLEGT)) [35, 36] (blue box in Fig. 1).

There is a growing focus on NCS interventions (alongside integrated actions) as a priority approach for nations to address drivers of environmental degradation and inequity in the pursuit of sustainable development [37]. Conversely, there are widespread concerns that rigid, blanket approaches to environmental protection and restoration will be detrimental to the ability of IPLCs to exercise and retain rights to traditional lands and resources, and potentially result in negative tradeoffs for human well-being [38–40]. For example, while the importance of land tenure security for achieving co-benefits for people and nature is well-documented [41, 42]—within the context of addressing deforestation, the nature of the links between tenure, economic well-being, and forest condition and cover varies significantly [43, 44]. As such, many emphasize that NCS must pay explicit attention to issues of equity and land and resource tenure security and be integrated with actions that are people-centered (e.g. blue box actions). While these actions are critical, in this map, we only examine them within the context of studies that aim to observe or measure the impact of the NCS interventions on climate change mitigation outcomes.

In this mapping work, we have chosen to focus on how these interventions link to changes in GHG emissions, carbon storage, and/or carbon sequestration through changes in land and management practices and changes in LULCC patterns. Within this conceptualization, we consider changes to land and management practices (e.g. adoption) and LULCC as intermediate outcomes along the pathway to climate change mitigation.

However, implementation of NCS does not occur in a vacuum and proper planning, design, implementation, and evaluation of interventions will need to take into consideration the co-impacts that an NCS intervention may have on biodiversity, ecosystems, and human communities. NbS interventions (of which NCS is a subset) for adaptation and resilience, individually, and as a whole, have been the subject of existing syntheses (e.g. [11, 12]) which represent a rich resource for contextualizing our findings from this primary mapping effort to better understand the distribution of studies that examine a combination of climate change mitigation and biodiversity, ecosystem, and/or socio-economic impacts within the broader context of existing syntheses in the space. Therefore, to better describe the state of evidence on co-impacts, we employ two approaches. First, *within* the evidence base on the links between NCS and our primary outcomes of interest (dark grey boxes in Fig. 1)—we will examine the extent to which studies *also* measure and assess socioeconomic and biodiversity/ecosystem outcomes (light grey boxes) alongside the primary outcomes of interest. We do not include primary studies that look *only* at the impact of NCS interventions on socioeconomic outcomes and/or biodiversity and ecosystem outcomes without measuring climate change mitigation and/or LULCC outcomes, given the resources available for this study. Second, as part of this systematic map effort, we will undertake a “mega-map” of existing systematic maps, reviews, and meta-analyses [45, 46] on the links between NCS and biodiversity, ecosystem, and socio-economic outcomes using the same Population (P) and Intervention (I) elements as this primary map (see Additional file 1 for full mega-map protocol details).

### Impacts across the carbon cycle

Soil organic matter plays an essential role in the global carbon cycle as well as providing key ecosystem services such as retention of water and nutrients which in turn support plant productivity [47]. Soil organic matter is composed of soil microbes (e.g. bacteria and fungi), decaying organic matter, and the byproducts of decomposition [48]. The level of carbon stored in soil is related to the volume of organic matter and is usually measured as soil organic carbon (SOC). Soil organic carbon levels are determined by the rates of photosynthesis, respiration, and decomposition of aboveground biomass. For example, aboveground plants fix atmospheric CO_2_ through photosynthesis and carbon enters the soil through the growth and death of roots as well as the deposition of leaf litter and other aboveground organic material [49]. Carbon can also be released from soils through decomposition where CO_2_ is released from microbial respiration (or ‘soil respiration’). Carbon can also be released as emissions to the atmosphere through soil disturbance (e.g. in agricultural activities and LULCC) [47].

This study focuses on the impacts at the first point where carbon is fixed into a terrestrial ecosystem (i.e. through aboveground biomass) and the last point where it exits (as emissions)—thus capturing the primary ways in which carbon storage, sequestration, and emissions are measured for tracking progress to climate change at scale. We focus on mitigation outcomes for emissions and carbon storage and/or sequestration within aboveground biomass in tropical and subtropical terrestrial ecosystems. While we recognize the significant contribution of storage and sequestration in soil carbon, given the time and resources available for this review, we are not including articles that *only* examine outcomes belowground (e.g. soil organic carbon or belowground biomass) (see Relevant Outcomes below). As belowground mechanisms of carbon storage and sequestration are also important, we will *also* code articles that measure impacts belowground in addition to the outcomes two listed above, thereby capturing the subset of articles that provide more comprehensive assessments (both above- and belowground outcomes).

## Methods

The methodology for building this systematic map includes three phases: (1) establishing key search terms to capture the concepts identified in the conceptual framework; (2) developing the scope and strategy of the search and inclusion/exclusion criteria; and (3) developing the scope and extent of a coding framework to extract relevant information to characterize the evidence base.

### Searching for articles

A comprehensive search will be undertaken to best capture an unbiased representation of existing literature related to our research question.

#### Language

Given available resources for this systematic map, we only search in English. We recognize that this may bias our systematic map as there may potentially be relevant literature in other languages [50]. Therefore, while we narrow our search to English, we will work with our review team to assess relevance of recovered studies in French, Spanish, and Portuguese.

#### Search terms

We compiled a set of search terms relevant to different components of the research question (Additional file 2). This string of terms was scoped in the Web of Science Core Collection to examine sensitivity given alternate terms and wildcards, as well as using input from our stakeholder team.

*Population*

(forest OR woodland OR meadow OR pasture OR agricultur* OR rangeland OR grassland OR mangrove OR tree OR cropland OR grazing OR land OR ecosystem OR landscape OR rice OR tropic*).

**AND**

*Intervention*

(restoration OR reforestation OR afforestation OR replanting OR rehabilitation OR enrichment OR "tree islands") OR ("rice production" OR "rice intensification" OR "rice cultivation" OR "community forest" OR "community forests" OR "community forestry" OR "shade grown" OR "climate-smart" OR "pasture management" OR "cover crop" OR "cover crops" OR "nutrient management" OR agroforestry OR agroforest OR silvopastor* OR silvopastur* OR silvo-pastor* OR silvo-pastur* OR agro-ecolog* OR agroecolog* OR "conservation agriculture" OR "tree planting" OR fencing OR exclosure OR ((partial OR selecti* OR gap OR retention) NEAR/3 (felling OR cutting OR harvest*)) OR "grazing management" OR "active management" OR "salvage logging" OR "reduced-impact logging" OR "alley cropping" OR "fire management" OR plantation OR "forest management" OR "manure management" OR ((crop OR cropland) NEAR/2 management) OR windbreaks OR thinning) OR ("protected area" OR "protected areas" OR ("Indigenous Peoples" OR "Indigenous communities" OR "Indigenous groups") OR "national park" OR "concession" OR "buffer zone" OR "sacred groves" OR "sacred forests" OR "sacred forest" OR "sacred grove" OR (protection NEAR/2 (forest OR landscape OR grassland))) OR ("land stewardship" OR "natural climate solutions" OR "natural climate solution" OR "ecosystem-based adaptation" OR "carbon forestry" OR "payments for ecosystem services" OR "payments for environmental services" OR "PES" OR "REDD" OR "REDD + " OR "Reduced Emissions from Deforestation and Degradation" OR "sloping land conversion" OR "cropland to forest").

**AND**

*Outcome*

("land use change" OR "land-use change" OR "land conversion" OR "forest conversion" OR "grassland conversion" OR deforestation OR "land cover" OR "forest cover" OR "vegetation cover" OR "habitat cover" OR "tree cover" OR (clearing NEAR/4 (forest OR land)) OR ((diversity OR composition OR recovery OR succession) NEAR/1 (tree OR forest)) OR ((biomass OR biomasses) NEAR/2 (tree OR shrub OR woody OR aboveground OR above-ground OR recovery OR living)) OR (degradation NEAR/2 (forest OR grassland)) OR ((climate OR carbon OR CO2 OR GHG OR "greenhouse gas") NEAR/3 mitigat*) OR ((carbon OR CO2) NEAR/2 (sequestration OR balance OR accounting OR storage OR emission OR sink OR stock OR fixation OR density)) OR (("greenhouse gas" OR GHG) NEAR/2 (emission OR avoid* OR reduc*)) OR aboveground OR above-ground).

**AND**

*Outcome adjacent*

(impact OR effect* OR evaluat* OR empiric* OR assess*).

**NOT**

(Canada OR "British Columbia" OR Europe OR Sweden OR Norway OR Finland OR Scandinavia* OR Mediterranean OR Chile OR "United Kingdom" OR Korea OR Pakistan OR Russia OR Denmark OR England OR Wales OR Ireland OR Scotland OR "integrated water resource management" OR European OR Spain OR Spanish OR Alabama OR Alaska OR Arizona OR Arkansas OR California OR Colorado OR Connecticut OR Delaware OR Florida OR Georgia OR Idaho OR Illinois OR Indiana OR Iowa OR Kansas OR Kentucky OR Louisiana OR Maine OR Maryland OR Massachusetts OR Michigan OR Minnesota OR Mississippi OR Missouri OR Montana OR Nebraska OR Nevada OR "New Hampshire" OR "New Jersey" OR "New Mexico" OR "New York" OR "North Carolina" OR "North Dakota" OR Ohio OR Oklahoma OR Oregon OR Pennsylvania OR "Rhode Island" OR "South Carolina" OR "South Dakota" OR Tennessee OR Texas OR Utah OR Vermont OR Virginia OR Washington OR "West Virginia" OR Wisconsin OR Wyoming OR "north america" OR "north american" OR Albania OR Andorra OR Armenia OR Austria OR Azerbaijan OR Belarus OR Belgium OR Bosnia and Herzegovina OR Bulgaria OR Croatia OR Cyprus OR Czechia OR Estonia OR France OR Germany OR Greece OR Hungary OR Iceland OR Italy OR Kazakhstan OR Kosovo OR Latvia OR Liechtenstein OR Lithuania OR Luxembourg OR Malta OR Moldova OR Monaco OR Montenegro OR Netherlands OR Poland OR Portugal OR Romania OR Russia OR San Marino OR Serbia OR Slovakia OR Slovenia OR Switzerland OR Turkey OR Ukraine OR "Vatican City" OR Alberta OR "British Columbia" OR Manitoba OR "New Brunswick" OR Newfoundland OR "Northwest Territories" OR "Nova Scotia" OR Nunavut OR Ontario OR "Prince Edward Island" OR Quebec OR Saskatchewan OR Yukon OR Labrador).

**NOT**

TI = ("modelling" OR "modeling").

#### Comprehensiveness of the search

We tested the comprehensiveness of the search string by testing it against a library of 30 relevant articles compiled by the review and stakeholder team and from backwards citation chasing (Additional file 3). We refined the search terms until we recovered all items in the test library that were indexed in Web of Science. We explicitly tested the impact of using NOT terms on the comprehensiveness of our search. With careful development and testing, the use of NOTs can improve the efficiency and precision of a search strategy, particularly when dealing with large corpora (e.g. [51–53]). We tested the likelihood that the use of NOTs would result in a significant portion of missed citations using a random sample and a relevance ranked sample of studies (n = 500 citations) that would have not been captured in a search strategy including NOT terms. We found that < 1% of recovered search results were ultimately relevant. While this percentage is very low, we account for potentially missed studies by searching other publication databases, using backward citation chasing, as well as an extensive grey literature search.

#### Searching the literature

Search for relevant published and unpublished literature, including both peer-reviewed and grey reports, will be conducted from the following sources: (1) within bibliographies of relevant reviews; (2) online publication databases, and (3) organization websites and repositories. This systematic map will begin with a rapid search for relevant reviews within the scope of the research question that document and report their search strategy and provide a list of included articles in their review. These reviews will be the basis for backwards citation chasing as a search strategy for identifying an initial pool of potentially relevant sources (see Additional file 2 for protocol). Citation chasing is a search approach that uses a list of relevant studies as the source within which to identify relevant articles. *Backwards citation chasing* involves looking at the citations that relevant articles cite in their work and screening *those* citations for inclusion in this synthesis. Starting a search strategy using backwards citation chasing allows one to identify an initial evidence base that is more likely to be relevant. This can help refine methods and train machine learning models to sort through search results more quickly and efficiently during the screening process. Specifically, we conduct the following searches:

1. *Backwards citation searches* This map will include a subset of data specifically pertaining to the review question derived from the bibliographies of 39 relevant reviews identified through systematic searches in publication databases (see searching details and bibliography in Additional file 4).
2. *Publication database searches* We will search Web of Science (Core Collection 1900-Present, 10 indices), Environment Complete (EBSCO). Selection of these databases was based upon previous systematic evidence syntheses on related topics (e.g. [20, 54]). We will search these databases for studies published prior to 2021.
3. *Topical databases and organization searches* Targeted searches of specialist websites and databases will be conducted, in particular, of established online repositories of impact evaluations, project and program evaluations, and systematic evidence syntheses on related topics to our research question (see list in Additional file 5). The first 100 results will be screened per website/database.
4. *Stakeholders and topic experts* Key informants and the broader research community will be contacted for relevant literature for screening and inclusion through electronic means (e.g. email, social media).

#### Reference management

We will use Zotero (www.zotero.org) to organize and store citations along the stages of this synthesis as it is open access and can be used across the review team. We will remove duplicate citations in Endnote (www.endnote.com) across searches, matching based on title (we use Endnote because of their functionality for de-duplication). The online synthesis platform colandr (www.colandrapp.com) will be used to screen citations at title and abstract. Colandr is an online, open-access evidence synthesis platform that has machine learning and text mining features to help improve the efficiency of screening and data extraction [54]. We will use the relevance ranking function to assess when it is likely that we have captured the majority of relevant citations. Based on other reviews that have employed machine learning functions to identify a stopping point [55], we will stop screening when we reach a 5% inclusion rate for every 300 citations screened (in order of ranked relevance). We will then spot check a random sample of the remaining citations to determine whether we should continue screening. We will conduct full text screening and data extraction in Knack, a relational database platform (https://www.knack.com) that can be used collaboratively across a team.

## Article screening and study eligibility criteria

### Screening process

Search results will be reviewed first at title and abstract to determine inclusion or exclusion (Table 1). Following a training set of studies screened by all reviewers, inter-rater reliability will be calculated using a Kappa statistic for all studies in the training set. Double screening will be conducted for 15% of articles at title and abstract and 10% at full text. Consistency checking will be conducted using a two-step, double-blind method employed within colandr, and inconsistencies will be discussed and reconciled across the team. Reviewers will not screen nor code articles that they are authors on.

**Table 1:** Summary table of draft inclusion and exclusion criteria (please refer to following sections and Additional files for details and rationale)

	Included	Excluded
**Population**	Tropical and subtropical terrestrial ecosystems	Marine, freshwater, coastal, and inundated ecosystems (e.g. peatlands, wetlands,except for mangroves) Urban and peri-urban settings
**Intervention**	Land stewardship interventions that aim to protect, manage, or restore existing natural terrestrial ecosystems Land stewardship interventions that aim to create or manage new ecosystems (e.g. afforestation, plantation forests, replanting with non-native plants, constructed ecosystems, artificial grasslands, natural/green infrastructure) in non-urban/non-peri-urban areas Interventions that aim to promote and implement sustainable and/or climate-smart agriculture, grazing, and agroforestry management and practices. Sustainable agricultural intensification within the bounds of climate-smart agriculture intended to reduce deforestation and land conversion	Effectiveness of existing ecosystems (without an intervention) Hybrid natural/engineered interventions Effectiveness of complementary interventions (e.g. training, capacity building, governance, equity, incentives, policies, monitoring, and enforcement) without explicit tie to land stewardship intervention Intensification of forestry and grazing activities—that may be intended to increase productivity while minimizing land use change (switching from one land type to another) or land use expansion
**Study type + comparator**	Studies that aim to measure or observe change in included outcomes as a result of the intervention Case studies, case reports, non-experimental, quasi-experimental or observational, and experimental study designs, systematic maps, and reviews	Studies that model or predict change to outcomes as a result of the intervention Modeling studies, opinions, editorials, non-systematic reviews or maps
**Primary outcome**	**Environmental outcomes directly related to GHG emissions** This includes *direct measures of GHG emissions* (as expressed as equivalent metric tons of CO_2_) and *carbon storage and sequestration aboveground* **Environmental outcomes indirectly related to change in mitigation** This includes intermediate (or “proxy”) outcomes related to *changes in aboveground land cover and condition* (e.g. change in biophysical characteristics of earth’s surface, including the distribution of vegetation and other aboveground physical features of the land, diversity, survival and growth of habitat forming species) and *changes in aboveground land use* (e.g. changes in the way that land is used by humans—e.g. land conversion, land sparing)	Studies that only examine changes to socioeconomic outcomes (including downstream impacts from changes to ecosystem service delivery such as productivity, crop yield, fodder) and/or biodiversity/ecosystem outcomes WITHOUT looking at one of the two outcome categories in the column to the left as well Ecological adaptation and resilience which do not link to the two outcomes to the left
**Secondary outcomes** (measured alongside with the primary outcomes above within included studies)	**Carbon storage and sequestration belowground** which includes changes to soil organic carbon **Land and forest management practice outcomes** *Adoption or uptak*e of land or forest management practices, or *agricultural practice or technologies* **Socioeconomic outcomes** which could include changes to economic and material well-being (incl. Income, jobs), perceived socioeconomic and/or poverty status, safety and security (incl. Vulnerability or risk to impacts of climate change (like floods, droughts, fires, temperature), rights and empowerment, cultural and spiritual well-being, sense of place, food security, health, nutrition, this includes elements of provisioning and cultural ecosystem services **Biological and ecological outcomes** which include changes to population abundance, dynamics, range and habitat extent and quality, connectivity, biodiversity. This includes elements of regulating and supporting ecosystem services (nutrient cycling, soil formation, primary productivity, climate, flood, temperature regulation)	

### Eligibility criteria

Eligibility criteria were developed collaboratively with the review team and in consultation with the advisory group. In cases where reviewers find there is insufficient information to make an informed decision on inclusion at title and abstract stage, the citation will be included to check at full text. To be included in the map, articles must fulfill the criteria outlined in Table 1. All exclusion reasons will be recorded at title and abstract and at full text and made available.

### Inclusion criteria

To be included in the systematic map, articles must meet the criteria outlined below.

#### Relevant population(s)

We focus specifically on tropical and subtropical terrestrial ecosystems as they not only have significant potential for addressing climate change through NCS [9, 10] but also are regions facing rapid land use change [56]. Given that examining all terrestrial ecosystems would be too large of a scope for the resources available, this systematic map examines tropical and subtropical forests, grasslands (incl. savannas and shrublands), and mangrove habitats as described using the terrestrial ecoregion classification system [57]. We recognize that inundated landscapes (e.g. marshes, peatlands, swamps, bogs, etc.…) have tremendous carbon storage potential, particularly belowground [58, 59]. However, due to time and resource constraints, we limit this effort to primarily aboveground ecosystems. While we recognize there are many existing classifications of terrestrial ecosystems, we use Dinerstein et al.’s [57] ecoregion classification as it is widely used by organizations working in natural climate solutions (e.g. NGOs, implementation agencies). We include the following five biomes:

- Tropical and Subtropical Coniferous Forests
- Tropical and Subtropical Dry Broadleaf Forests
- Tropical and Subtropical Grasslands, Savannas & Shrublands
- Tropical and Subtropical Moist Broadleaf Forests
- Mangroves

#### Relevant primary intervention(s)

NCS encompasses actions from across the nature conservation, natural resource management, and agricultural sectors. We synthesize existing typologies of actions and practices from across all three sectors [2, 11, 20, 54, 60–62] to frame NCS interventions for this systematic map. Specifically, we aggregate actions across three broad themes—protection, management, and restoration (Table 2, Additional file 6). We disaggregate between management of agricultural areas and management of forest and other land use areas. We focus on interventions that explicitly target or are hypothesized to have impacts on GHG emissions and/or carbon storage/sequestration. In addition, while mitigation options within AFOLU sectors primarily focus on supply-side actions such as those within NCS, they also recognize the potential contribution of food/dietary/behavior demand-side options for climate change mitigation (e.g. [13]). We recognize the importance of demand-side opportunities, however, these are beyond the scope of this map given available resources.

**Table 2:** Typology of primary natural climate solution interventions (detailed typology in Additional File 6)

Category	Definition
Protection	*Establishing or expanding measures of protection for natural or semi-natural ecosystems for the purposes of conserving/regulating ecosystem services and preventing the loss of natural landscapes/resources. Land or resource use is either fully restricted or significantly regulated. In particular, actions in this space intended to prevent conversion of forest or grasslands to tilled croplands and other intensive land uses (e.g. residential, mining)* *Examples include protected areas, parks, indigenous territories*
Forest and Other Land Use Management	*Actions directed at managing existing natural or semi-natural ecosystems OR created ecosystems for either the purposes of conserving/regulating ecosystem services and natural landscapes and/or providing sustained natural resources for use. In the context of NCS, management actions can avoid GHG emissions or enhance carbon sinks on working lands through improved management practices that do reduce existing yield* *Examples include forest management, forestry, grasslands management, climate-smart forestry, reduced impact logging, agroforestry, Ramsar sites (for mangroves). This would include management actions to restore carbon stocks in existing production lands*
Agricultural Management	*Agricultural systems that increase food security in the face of climate change, enhance adaptive capacity of farmers to the impacts of climate change, and mitigate climate change where possible. Climate-smart agriculture (CSA) approaches should (1) address climate or weather related risk (both extreme and slow-onset events) while improving food security in the short and long term, (2) provide at the minimum two benefits out of productivity, resilience, and mitigation, and (3) be socially and culturally appropriate for the area where they are being practiced. These technologies are typically accompanied by actions to improve enabling conditions—e.g. infrastructure development, social safety nets. To be included in this map, we focus on climate-smart agricultural practices that aim to reduce GHG emissions and/or store carbon aboveground (i.e. excluding measures solely targeted at soil carbon sequestration)* *Examples include conservation agriculture, nutrient management, improved rice cultivation, agroecological practices, agroforestry, silvopastoralism, livestock and grazing management, and manure management*
Restoration	*Re-establishing, enhancing, or establishing ecosystems to return them to natural or semi-natural states for the purposes of conserving/regulating ecosystem services and expanding the spatial extent of natural landscapes that have been lost due to previous human activity. Includes actions to create new ecosystems in place of a naturally occurring one or where one does not exist* *Examples include reforestation, afforestation and passive restoration*, *and other approaches following the principles of Forest Landscape Restoration (FLR)*

#### Relevant comparator(s)

Only studies that employ a valid comparator will be included. This does not mean that only quantitative impact evaluations based on experimental designs will be included, but that studies should aim to measure change that results from an intervention(s). As this study is attempting to describe the state and characteristics of the evidence base, particularly in relation to how the impact of these interventions is being evaluated and where key gaps in evaluation effort may exist, we included a broad range of comparator and data types to provide insight on overall study quality.

Comparators will be classified as temporal, spatial, or between groups. Temporal comparators examine effects over time including before and after implementation, interrupted or continuous time series and reported/perceived changes (particularly in the case of change in adoption or uptake of land and agricultural practices). Spatial comparators that compare effects between sites over distance (e.g. near vs. far, linear distance) will be included. Lastly, between group comparators compare effects between populations either of species/types of ecosystems or humans related to the intervention will be included. This includes comparisons between presence/absence of an intervention as well as across sites within an intervention or across interventions.

#### Relevant study type(s)

We will include primary research articles in English meeting the criteria outlined below:

- Non-experimental, quasi-experimental, and experimental study designs that use quantitative, qualitative, or a combination of data types
- Systematic reviews and syntheses (e.g. systematic maps, evidence gap maps, meta-analyses) that describe the methods used for search, data collection, and synthesis as per the ‘green’ level standards for the Collaboration for Environmental Evidence Systematic Appraisal Tool (CEESAT) [63]

We will exclude the following:

- Theoretical or modeling studies, editorials and commentaries will be excluded
- Literature reviews which do not describe methods used for search, data collection, and synthesis (e.g. as per ‘red’ or ‘yellow’ level standards for CEESAT)

#### Relevant outcome(s)

We will include studies that assess the impact of NCS interventions on climate change mitigation. We focus on mitigation outcomes for GHG emissions and carbon storage and/or sequestration within aboveground biomass in five tropical terrestrial biomes. As belowground mechanisms of carbon storage and sequestration are important, we will code for articles that also measure impacts belowground in addition to the two listed above.

We use categories and subcategories of outcomes as outlined in [20, 64], adapted to reflect both direct and proxy measurements of change in GHG emissions and carbon storage and sequestration (Table 3, Additional file 7). We will not look at impacts on productivity or yield for aboveground resources unless the measure of biomass is treated as a proxy for climate change mitigation outcomes. We will not include articles that use perception data as a measurement for these outcomes.

**Table 3:** Typology of outcomes (see Additional file 7 for detailed typology)

Category	Subcategory	Definition
Climate change mitigation	Greenhouse gas emissions	Change in GHG emissions (including nitrous oxide (NO_2_), methane (CH_4_), and carbon dioxide (CO_2_)) as measured in equivalent metric tons of CO_2_ as a result of intervention
	Carbon storage and sequestration	Measure of change in aboveground biomass carbon stocks
	Land condition	Measures of change in characteristics of existing ecosystems, including composition, structure, or function of that ecosystem that affects its carbon storage potential
	Land cover	Any measure of change in vegetation cover, including extent of vegetation maintained, recovered/regenerated, deforested, or converted to another land type. Also includes outcomes related to wood extraction from forests for fuel (as a proxy for deforestation)

### Study validity assessment

Due to the volume of studies that are likely to be encountered and the wide breadth of study designs and comparators considered, we will not be critically appraising the validity or robustness of included articles. We recognize that this will limit interpretations from the resulting evidence map and potentially the utility of the map for end users. As such, we will consider this limitation appropriately and fully within the analysis portion of this study. However, we will code aspects of study design (e.g. type of design, comparator types, etc.…) that will provide an initial idea of overall study validity within the evidence base. Furthermore, we will assess the distribution of study designs employed within the resulting evidence map in order to provide guidance on how to assess study quality in the future.

### Data coding strategy

We will use a standardized data coding form to extract descriptive meta-data from all studies meeting our inclusion criteria. Data extracted from each study will include:

- Bibliographic details (such as author affiliations)
- Intervention type and details (as in typology above)
- Any complementary interventions implemented alongside or in concert with the intervention (and details) (see below Table 4)
- Study location, scale, design, and comparator details
- Outcome type and details (including any disaggregation in measurement and indicators used)
- Details on adjacent outcomes evaluated in addition to the primary outcomes of interest (socioeconomic and/or biological/ecological outcomes, belowground outcomes, changes to practice/adoption/uptake) (see below, Tables 5–7).

**Table 4:** Complementary intervention typology (see Additional File 6)

Category	Definition
Policies, laws, mandates, and regulation	Actions to develop, change, influence, and help implement formal legislation, regulations, and voluntary standards aimed at supporting climate change mitigation actions
Training, technical support, and capacity building	Actions to build capacity to do better conservation including developing partnerships and institutions as well as improving understanding and skills
Good governance and securing rights	Actions taken to define and secure rights to resources for and by local actors, build local capacity for management and participation and empowerment in decision-making, improving and strengthening governance structures and processes to ensure fair and equitable participation, inclusion, transparency, and accountability in management of natural resources and ecosystems
Livelihood, economic & other incentives	Actions to use economic and other incentives to influence behaviour around climate change mitigation actions

**Table 5:** Socioeconomic outcomes typology (see Additional File 7 for detailed typology)

Category	Definition
*Economic well-being*	This encompasses the living standards of basic life including both economic and material necessities Economic living standards: Income, employment, employment opportunities, wealth, poverty, savings Material living standards: Material assets owned, basic infrastructure (electricity, water, telecommunication and transportation), shelter, resource use (this can include sustainable use/harvest)
*Health*	Any component of individual mental or physical health or access to health services
*Safety and security*	Covers any component of physical security (threat to personal body and community sense of safety), and resilience and/or adaptive capacity to respond to changing environments and shocks
*Rights and empowerment*	Structures and processes for decision-making that include both formal and informal rules and ability of individuals and groups to be heard and participate in formal and informal decision-making processes. Includes changes to de jure and de facto bundle of rights to land and resources and ability to exercise, as well as tenure, land, and carbon rights
*Education and skills*	Includes both formal and information education and training outcomes as well as educational infrastructure. Includes changes in awareness of climate change and/or environmental issues
*Social capital*	Includes measures of the networks of relationships among individuals and groups that live and work within a particular society and include relations to 'external' groups (such as foreign implementers) as well as the 'state' (formal government at various scales)
*Culture*	Cultural, societal, and traditional values related to natural resources and nature to an individual, group, and/or community and traditional and Indigenous knowledge, activities, and practices
*Agricultural productivity*	Agricultural productivity is typically measured in crop yield—the harvested production per unit of harvested area for crop products [67]. We also include measures of crop growth and survival in this category

A complementary codebook will be created to explain the scope of each question/field in the data coding form (Additional file 8). The review team will conduct a pilot with a small subset of articles to ensure consistency and to resolve any issues or ambiguities in interpretation. Depending on the volume of articles included for coding, we will conduct side-by-side double coding of data of 10% of total included articles. For the remaining articles, random spot checks will be conducted to ensure consistency between reviewers. We will heuristically measure consistency using percent disagreement of spot-checking with the primary reviewer.

### Complementary interventions

Complementary interventions are activities that aim to address the enabling conditions and/or contextual factors that are critical for the success of the primary intervention. It is well established that addressing global environmental challenges, such as climate change, will require multisectoral and integrative solutions that aim to influence change at the system level [65]. We use the typology detailed in Table 4 to code for complementary interventions when they are present.

### Presence of biological, ecological, and/or socioeconomic outcomes

In addition to mitigating climate change, NCS interventions can likely have immediate and downstream effects on socioeconomic, biological, and ecological domains. For example, adoption of climate-smart agricultural practices could have immediate impacts on farmer livelihoods (e.g. additional resources spent on new technologies or materials, temporary changes in yields due to new practices) as well as eventual downstream impacts (e.g. improved resilience to climate change impacts, potential changes in patterns of production and consumption). Similarly, efforts such as restoration of degraded ecosystems will have immediate impacts on abundance of certain species, as well as downstream impacts on ecosystem function. We recognize that examining how NCS impacts these socioeconomic and biological domains is critical to understanding the systems-level impacts of these interventions—however, due to the volume of likely resources on these topics, including these outcomes as part of our PICO framework is not feasible.

We will, however, examine whether articles that examine impacts on practice and/or climate change mitigation outcomes also examine socioeconomic, biological, and/or ecological outcomes. This approach will allow us to assess the extent of the evidence base that examines potential co-benefits of NCS interventions for climate mitigation and biodiversity or socioeconomic outcomes, as minimizing tradeoffs and maximizing co-benefits is critical to meeting multiple global climate targets and Sustainable Development Goals. We take a holistic view of socioeconomic outcomes that reflect impacts at both the individual, group, and community levels (Table 5). Similarly, we frame the biological and ecological outcomes along the lines of GEOBON’s ‘essential biodiversity variables’ framework (species, community, ecosystem) [66] to better align categories from this map with broader efforts within the biodiversity monitoring and evaluation community for data standardization (Table 6). Given the central role that NCS generally play for supporting ecosystem services, we integrate elements of provisioning and cultural services within socioeconomic outcomes (including agricultural productivity) and regulating and supporting services within biological and ecological outcomes (Tables 5 and 6). Lastly, we also look at immediate impacts on adoption and/or uptake of practices as these are a critical intermediate outcome within the causal pathway to climate change mitigation outcomes (Table 7).

**Table 6:** Biological and ecological outcomes typology (see Additional file 7 for detailed typology)

Category	Definition
*Population/species*	Outcomes focused on change in populations of individuals or populations within species
*Ecological community*	Outcomes focused on change in community conditions
*Ecosystem function*	Outcomes focused on change in ecosystem processes and conditions, includes regulating ecosystem services (e.g. mediation of waste, toxins, and other nuisances; mediation of flows; maintenance of physical, chemical, and biological conditions including life cycle, disease, soil formation, water and climate regulation)

**Table 7:** Adoption and behavior outcomes typology (see Additional file 7 for detailed typology)

Category	Subcategory	Definition
Land and agricultural management practice	*Acquisition of knowledge/skills*	Changes in knowledge of new practices and technology, for or of environmental impact of existing practices/technology
	*Adoption of new practices or technology, cessation of current practices*	Adoption of technology or sustainable agricultural practices such as, use of fertilizer and pesticide, improved crop varieties. Abandonment of current practices or choosing to do nothing (e.g. natural regeneration on abandoned land)

### Measuring belowground carbon storage and sequestration

Soil carbon storage and sequestration are important components of the mitigation potential of NCS [68]. We will code articles if they *also* measure impacts on soil carbon/belowground alongside changes in aboveground storage/sequestration and/or emissions.

## Study mapping and presentation

The final dataset will be formatted for statistical analysis in R (https://www.R-project.org/) to enable us to summarize key characteristics and trends. We will summarize the descriptive characteristics of the included studies according to the population, interventions, comparators, biome/ ecoregion, study designs and outcomes and present in visual frequency graphs. We will generate visual representations of the distribution of the evidence base in terms of geographic distribution, as well as types of interventions and outcomes assessed in the current literature (‘heatmaps’). We will use this to identify and characterize the presence of different types of evidence gaps; for example, ‘absolute’ evidence gaps (where there is no literature evaluating impacts), evaluation gaps (where there is some volume of literature but insufficient quasi-experimental or experimental impact evaluations), and synthesis gaps (where there are literature documenting impacts but a lack of high-quality systematic reviews synthesizing this information). In particular, we will describe the current state and distribution of the evidence base in regards to the number of studies that look at secondary outcomes and compare that to the distribution of studies synthesized in the ‘mega-map’ effort.

Trends and patterns in the data along with relevant insights for policy, practice and research will be summarized in a narrative report in addition to the graphical display. Final data on excluded literature, included literature, and associated meta-data will also be made available in an online open source repository, and code for analysis will be made available on Github.

## Supplementary Information

**Additional file 1:** Protocol for mega-map.

**Additional file 2: **Search strategy 10–2.

**Additional file 3: **Test library.

**Additional file 4: **Protocol for backwards citation chasing.

**Additional file 5: **Grey literature sources.

**Additional file 6: **Intervention typology.

**Additional file 7: **Outcome typology.

**Additional file 8: **Codebook for data extraction.

**Additional file 9: **ROSES systematic map protocols checklist.

## Data Availability

All data generated or analyzed in this study are included in this article, additional files, and supplementary information.
