# Supplementary material for: What evidence exists on the links between natural climate solutions and climate change mitigation outcomes in subtropical and tropical terrestrial regions? A systematic map protocol
Source: Environ Evid. 2022 Apr 19;11:15. doi: 10.1186/s13750-022-00268-w (PMC9017726; doi:10.1186/s13750-022-00268-w)
Supplement: Supplementary file 1 — Additional file 1: Protocol for mega-map. [file 13750_2022_268_MOESM1_ESM.docx]

**Natural climate solutions**

*Mapping existing syntheses - protocol*

**Objective of this Mega Map:**

This study is a rapid effort to systematically map and describe the distribution of existing systematic maps, systematic reviews, and evidence gap maps on the links between NCS interventions and socio-economic and biophysical outcomes. This is complementary to a map that identifies, maps, and describes the evidence on the impacts of natural climate solution interventions on climate change mitigation outcomes. The objective of this “mega map” is to characterize the existing synthesized evidence base on socio-economic, biological, and ecological impacts to provide context for the findings of the main systematic mapping effort that is focused on impacts on (1) land and forest management practices and restoration practices (2) climate change mitigation outcomes in forest, agricultural, and grassland landscapes in tropical regions.

**Overarching Key Question:**

What is the evidence base for the links between natural climate solutions (NCS) interventions and impacts on socio-economic and biophysical outcomes in forest, agricultural, and grassland landscapes in tropical regions?

**Elements of the Primary Question:**

**Population**

- Terrestrial ecosystems and mangroves in tropical countries

**Intervention**

- Land stewardship interventions that aim to protect, manage, or restore existing natural terrestrial ecosystems
- Land stewardship interventions that aim to create or manage new ecosystems in non-urban/peri-urban areas
- Interventions that aim to promote and implement sustainable and/or climate-smart agriculture, grazing, and agroforestry management and practices.
- Studies covering passive restoration and conservation interventions

**Study type**

- Systematic maps and reviews, gap maps, or any literature review that clearly documents the search strategy and provides a list of included articles
- Review articles that aim to examine the link between an NCS intervention and one or more of the target outcomes

**Outcomes**

- Socio-economic outcomes (including provisioning and cultural ecosystem services)
- Biological and ecological outcomes (including regulating and maintenance services)

**METHODS**

The methods for building a mega map of the distribution of existing systematic maps, systematic reviews, and gap maps on the links between NCS interventions and socio-economic and biophysical outcomes include two components: (a) establishing key search terms to identify possible maps and reviews; and (b) developing the scope of the search and inclusion and exclusion criteria.

**Search process**

*Search terms*

We compiled an initial set of English search terms relevant to the different components of the primary research question. We performed a scoping exercise using Web of Science Core Collections and swapping in and out individual terms to test sensitivity and specificity of the search. We developed a [test library](https://docs.google.com/spreadsheets/d/1l4F66v39fwm1ArwBQ7ooXqXw1EgJPPnrV_VO1oaAx-E/edit#gid=361379818) of 19 papers identified through initial searches and discussions with experts in the field to use as benchmarks in the scoping exercise and to help identify and refine key terms.

Our final search string includes terms for population, intervention, study type and biodiversity as well as human well-being outcomes.

**Terms in Finalized Search String**

***[Population]***

(forest OR woodland OR meadow OR pasture OR agricultur* OR rangeland OR grassland OR mangrove OR tree OR cropland OR grazing OR land OR ecosystem OR landscape OR rice OR tropic*)

***AND***

***[Intervention]***

(restoration OR reforestation OR afforestation OR replanting OR rehabilitation OR enrichment OR "tree islands") OR TS=("rice production" OR "rice intensification" OR "rice cultivation" OR "community forest" OR "community forests" OR "community forestry" OR "shade grown" OR "climate-smart" OR "pasture management" OR "cover crop" OR "cover crops" OR "nutrient management" OR agroforestry OR agroforest OR silvopastor* OR silvopastur* OR silvo-pastor* OR silvo-pastur* OR agro-ecolog* OR agroecolog* OR "conservation agriculture" OR "tree planting" OR fencing OR exclosure OR ((partial OR selecti* OR gap OR retention) NEAR/3 (felling OR cutting OR harvest*)) OR "grazing management" OR "active management" OR "salvage logging" OR "reduced-impact logging" OR "alley cropping" OR "fire management" OR plantation OR "forest management" OR "manure management" OR ((crop OR cropland) NEAR/2 management) OR windbreaks OR thinning) OR TS=("protected area" OR "protected areas" OR ("Indigenous Peoples" OR "Indigenous communities" OR "Indigenous groups") OR "national park" OR "concession" OR "buffer zone" OR "sacred groves" OR "sacred forests" OR "sacred forest" OR "sacred grove" OR (protection NEAR/2 (forest OR landscape OR grassland))) OR TS=("land stewardship" OR "natural climate solutions" OR "natural climate solution" OR "ecosystem-based adaptation" OR "carbon forestry" OR "payments for ecosystem services" OR "payments for environmental services" OR "PES" OR "REDD" OR "REDD+" OR "Reduced Emissions from Deforestation and Degradation" OR "sloping land conversion" OR "cropland to forest")

***AND***

***[study type]***

("systematic review" OR "systematic map" OR meta-analyses OR meta-analysis OR "evidence review" OR "gap map" OR "systematic literature" OR "systematic evidence")

***AND {***

***[biodiversity outcomes]***

(“species diversity” OR “species richness” OR biodiversity OR behavio* OR predat* OR richness OR environment* OR ecolog* OR composition OR function)

***OR***

***[human well-being outcomes]***

(“livelihood” OR rights OR (“well being”OR wellbeing) OR vulnerability OR “food security” OR “ecosystem service*” OR social OR cultural OR (“indigenous knowledge” OR “traditional knowledge” OR “traditional ecological knowledge” OR “local knowledge” OR “local ecological knowledge”) OR (health OR “health services”) OR productivity OR yield OR (economic or socioeconomic) OR (equity or justice))

**Searching the Literature**

In this project, we identified potentially relevant reviews from specialist databases, expert solicitation, and a targeted search within a bibliographic database.

Searches for relevant published academic and grey literature were performed in the English language with predefined search terms. The searches were conducted in (i) bibliographic databases and (ii) specialist databases, including online publication databases and organisational websites, and (iii) previous review of reviews. All database subscriptions were through Columbia University in New York City, US.

(i) Bibliographic databases

We searched the following databases for articles:

1. Web of Science Core Collections
2. Scopus
3. Environment Complete Columbia

(ii) Specialized databases/Journals

1. Campbell Collaboration
2. CEEDER

(iii) Previous reviews of reviews

This meta map examined a list of potentially relevant articles from a related review-of-reviews effort that covers the inclusion criteria for this meta-map and includes documents from the following sources: CEEDER, Environmental Evidence Journal, 3ie, and Environment Complete. The protocol for this review can be found (Supplementary Information 1).

**Criteria for inclusion**

*Inclusion criteria*

To be included in the review of reviews, studies must meet the criteria outlined below.

**Table 1.** Inclusion and exclusion criteria at title and abstract

|  | **Included** | **Excluded** |
| --- | --- | --- |
| **Population** | Terrestrial ecosystems in tropical countries  (Tropical & Subtropical Coniferous Forests; Tropical & Subtropical Dry Broadleaf Forests; Tropical & Subtropical Grasslands, Savannas & Shrublands; Tropical & Subtropical Moist Broadleaf Forests; Mangroves) | Marine, freshwater, coastal, and inundated ecosystems (except for mangroves)  Peatlands, wetlands,  Non-tropical countries [or regions, or terrestrial ecosystems]  Urban and peri-urban settings [need to define either or both of these…???] |
| **Intervention**  **Full typology** [**here**](https://docs.google.com/spreadsheets/d/1ASQwVH-AIB7qlXX6DTd7_MsMIndaWLtPoJClhaLmsvM/edit#gid=0) | Land stewardship interventions that aim to protect, manage, or restore existing natural terrestrial ecosystems  Land stewardship interventions that aim to create or manage new ecosystems (e.g. afforestation, plantation forests, replanting with non-native plants, constructed ecosystems, artificial grasslands, natural/green infrastructure) in non-urban/peri-urban areas  Interventions that aim to promote and implement sustainable and/or climate-smart agriculture, grazing, and agroforestry management and practices. Sustainable agricultural intensification within the bounds of climate-smart agriculture intended to reduce deforestation and land conversion.  Studies covering passive restoration **(limited to interventions within the last 20 years)** and conservation interventions (ie protected areas, reforestation/afforestation efforts) | Effectiveness of existing ecosystems (without an intervention)  Hybrid natural/engineered interventions including growing of biofuels and crops for alternative energy and biotechnology interventions (e.g. engineering trees)  Effectiveness of complementary interventions (e.g. training, capacity building, governance, equity, incentives, policies, monitoring and enforcement) without explicit tie to land stewardship intervention  Interventions that focus solely on belowground biomass (e.g. soil, watershed, submerged vegetation)  Intensification of forestry and grazing activities - that may be intended to increase productivity while minimizing land use change (switching from one land type to another) or land use expansion  Biochar |
| **Study type + comparator** | Systematic maps and reviews, evidence gap maps, or any literature review that clearly documents their search strategy and provides a list of included articles  Review articles must aim to examine the link between an NCS intervention and one or more of the outcomes included below | Primary studies, modeling studies, opinions, editorials, non-systematic reviews or maps, or any review that does not document their search strategy and does not provide a list of included articles  Protocols for reviews  Topic of review must be impact of intervention on outcomes |
| **Outcome(s)**  **Typologies**  [**here**](https://docs.google.com/spreadsheets/d/1XAvwnwtABM1g06Mj9f0tjMq480PVOtUiCwtpmstica4/edit#gid=0) | **Socio-economic outcomes (including provisioning and cultural ecosystem services)** which could include changes to economic and material well-being, perceived socioeconomic status, increased income, ecosystem services, rights and empowerment, cultural and spiritual well-being, aesthetic values, social relations, sense of place, cultural heritage, food security, agricultural productivity including yield, health, livelihoods, education, traditional or ecological knowledge, equity, and justice.  Resilience or vulnerability or risk of individuals or communities to impacts of climate change (e.g. floods, droughts, fires, temperature)  **Biological and ecological outcomes** which include changes to non-habitat forming species richness or diversity, biodiversity, behavior, interspecies dynamics (e.g. predation), population abundance and dynamics, species range, habitat extent and quality, etc… and **regulating and maintenance services** such as raw materials, energy, nutrient cycling, soil formation, primary productivity, climate, flood, temperature regulation, fire regulation, water quality and flows. | Climatic outcomes (e.g. precipitation, temperature, etc…)  Physical outcomes (e.g. evapotranspiration, water filtration, geomorphological outcomes, etc…)  Climate change mitigation outcomes (ones listed for main map)  GMOs, crop management  Land and forest management practice outcomes (e.g. adoption or modification of practices)  Environmental outcomes directly related to GHG emissions (direct measures)  Changes to land cover or quality (extent of tree and habitat cover, deforestation….)  Land condition (changes to habitat-forming species - trees, shrubs, and grasses) |

**Screening reviews at title and abstract**

We will use a decision tree approach for screening reviews for inclusion. We will have one decision tree for screening at title and abstract and one decision tree specifically for screening at full text (see [here](https://docs.google.com/presentation/d/13XpI7ZkdU4LUrM61xnillpF0sormI7G8oWhVwmo9his/edit#slide=id.gf4c29b2976_0_59) for both). Screening at title and abstract will occur in colandr - exclusion criteria correspond to those on the decision tree.

**Retrieving PDFs and reference management**

We will use Zotero to organize and store citations along the stages of this synthesis. Colandr (Cheng et al. 2018) will be used to screen citations at title and abstract and full text. A project workspace will be established to assist the research team in organizing and managing sources of evidence (i.e. where possible studies are located) and the screening and coding process.

**Screening at full text**

Full text inclusion criteria are the same as inclusion criteria at title and abstract; however, we will be specifically assessing whether the reviews:

- Include if it contains list of search terms AND where they searched
- Include if it contains list of included articles (beyond listing them within the references)

Full text screening and coding will be conducted on the Knack platform.

**Coding meta-data and assessing review reporting and conduct**

If an article is included at full text, we will code the following meta-data from each review article in order to characterize and describe the evidence base:

- Bibliographic information
- Study design information
- Intervention information
- Complementary intervention information
- Outcome information
- Quality and transparency of reporting of review methods (using CEESAT criteria)

We will use the following codebook to extract information from all included articles.

**Table 2.** Draft codebook

| **General** | | | |
| --- | --- | --- | --- |
| 0.1 | Article ID | Number | Unique ID - see complete list of articles for full text assessment |
| 0.2 | Name of Assessor | Text | Initials |
| 0.3 | Date of article download | Date | Day/Month/Year |
| 0.4 | Date of assessment | Date | Day/Month/Year |
| 0.5 | Name of second assessor (if verified) | Text | Initials |
| **Bibliographic information** | | | |
| 1.1 | Publication type | Select one | Book/book chapter, Conference proceedings, Peer-reviewed published article, Project report, Working paper, Other |
| 1.2 | Author(s) | Text | Authors of study |
| 1.3 | DOI | Text | DOI link for article |
| 1.4 | Year of publication | List | Year of publication |
| 1.5 | Title | Text | Title |
| 1.6a | Journal name | Text | Journal name |
| 1.6b | Volume | Text | Volume(Issue) |
| 1.6c | Page # | Text | Page numbers |
| 1.6d | Publisher | Text | Publisher |
| 1.6e | Publisher location | Text | Publisher location |
| 1.7 | Affiliation of author(s) | Text | Lead author only |
| 1.8 | Affiliation type | Select many | Academic, Public sector, Research Institute, Consultant, Non-profit, Private sector/Industry (can select more than one) |
| 1.9 | Funding source | Text | Source: Acknowledgements section |
|  |  |  |  |
| **Basic information on study** | | | |
| 2.1 | Study objective | Text |  |
| 2.2 | Stated review type | Text |  |
| 2.3 | Source of articles | Text | Where authors obtained articles from (e.g. Web of Science, Scopus, expert solicited, etc...) |
| 2.4 | Publication years covered by review | Text |  |
| 2.5 | Number of included articles | Text |  |
|  |  |  |  |
| **PICO** | | | |
| 3.1 | Description of population(s) | Text |  |
| 3.2 | Biome category | Select many | Forest, Grasslands, Mangroves, Agriculture |
| 3.3 | Scale of review | Select many | Global, Regional, National, Subnational, Local |
| 3.4 | Country(ies) of study | Select many |  |
| 3.5 | Description of intervention(s) and any complementary action(s) | Text | Copy and paste in description from text |
| 3.6 | Intervention category(ies) | Select many | Choose from list in intervention typology |
| 3.7 | Complementary action category(ies) | Select many | Choose from list in complementary actions typology |
| 3.8 | Description of types of studies/comparators included | Text | Copy and paste in description from text |
| 3.9 | Description of outcome(s) | Text |  |
| 3.10 | Outcome type(s) | Select many | Choose from list in outcome typologies |
| **General findings** | | | |
| 4.1 | Report general findings/conclusions from review | Text | Copy and paste key findings/conclusions from the review |
| 4.2 | What would you cite this review for? | Text | Write in personal perspectives of what you would use this review for |
|  |  |  |  |
| **Review reporting and quality (Use CEESAT criteria)** | | | |
| 5.1 | 1.1 - Are the elements of the review question clear? | Select one | Red, Amber, Green, Gold |
| 5.2 | 2.1 - Is there an a-priori method/protocol document? | Select one | Red, Amber, Green, Gold |
| 5.3 | 3.1 - Is the approach to searching clearly defined, systematic and transparent? | Select one | Red, Amber, Green, Gold |
| 5.4 | 3.2 - Is the search comprehensive? | Select one | Red, Amber, Green, Gold |
| 5.5 | 4.1 - Are eligibility criteria clearly defined? | Select one | Red, Amber, Green, Gold |
| 5.6 | 4.2 - Are eligibility criteria consistently applied to all potentially relevant articles and  studies found during the search? | Select one | Red, Amber, Green, Gold |
| 5.7 | 4.3 - Are eligibility decisions transparently reported? | Select one | Red, Amber, Green, Gold |
| 5.8 | 5.1 - Does the review critically appraise each study? | Select one | Red, Amber, Green, Gold |
| 5.9 | 5.2 - During critical appraisal was an effort made to minimise subjectivity? | Select one | Red, Amber, Green, Gold |
| 5.10 | 6.1 - Is the method of data extraction fully documented? | Select one | Red, Amber, Green, Gold |
| 5.11 | 6.2 - Are the extracted data reported for each study? | Select one | Red, Amber, Green, Gold |
| 5.12 | 6.3 - Were extracted data cross checked by more than one reviewer? | Select one | Red, Amber, Green, Gold |
| 5.13 | 7.1 - Is the choice of synthesis approach appropriate? | Select one | Red, Amber, Green, Gold |
| 5.14 | 7.2 - Is a statistical estimate of pooled effect (or similar) provided together with measure of variance and heterogeneity among studies? | Select one | Red, Amber, Green, Gold |
| 5.15 | 7.3 - Is variability in the study findings investigated and discussed? | Select one | Red, Amber, Green, Gold |
| 5.16 | 8.1 - Have the authors considered limitations in the synthesis? | Select one | Red, Amber, Green, Gold |

We will use the following typologies to categorize interventions and outcomes

**Table 3.** Typology of natural climate solution interventions (see [here for detailed typology](https://docs.google.com/spreadsheets/d/1ASQwVH-AIB7qlXX6DTd7_MsMIndaWLtPoJClhaLmsvM/edit#gid=0))

| **Category** | **Definition** |
| --- | --- |
| Protection | *Establishing or expanding measures of protection for natural or semi-natural ecosystems for the purposes of conserving/regulating ecosystem services and preventing the loss of natural landscapes/resources. Land or resource use is either fully restricted or significantly regulated. In particular, actions in this space intended to prevent conversion of forest or grasslands to tilled croplands and other intensive land uses (e.g. residential, mining, etc…).*  *Examples include protected areas, parks, indigenous territories, etc…* |
| Forest and Other Land Use Management | *Actions directed at managing existing natural or semi-natural ecosystems OR created ecosystems for either the purposes of conserving/regulating ecosystem services and natural landscapes and/or providing sustained natural resources for use. In the context of NCS, management actions can avoid GHG emissions or enhance carbon sinks on working lands through improved management practices that do reduce existing yield.*  *Examples include forest management, forestry, grasslands management, climate-smart forestry, reduced impact logging, agroforestry, etc… This would include management actions to restore carbon stocks in existing productive land.* |
| Agricultural Management | *Agricultural systems that increase food security in the face of climate change, enhance adaptive capacity of farmers to the impacts of climate change, and mitigate climate change where possible. Climate-smart agriculture (CSA) approaches should (1) address climate or weather related risk (both extreme and slow-onset events) while improving food security in the short and long term, (2) provide at the minimum two benefits out of productivity, resilience, and mitigation, and (3) be socially and culturally appropriate for the area where they are being practiced. These technologies are typically accompanied by actions to improve enabling conditions - e.g. infrastructure development, social safety nets, etc… To be included in this map, we focus on climate-smart agricultural practices that focus on reducing GHG emissions and/or storing carbon aboveground (i.e. excluding measures solely targeted at soil carbon sequestration).*  *Examples include conservation agriculture, nutrient management, improved rice cultivation, agroecological practices, livestock and grazing management, and manure management* |
| Restoration | *Re-establishing, enhancing, or establishing ecosystems to return them to natural or semi-natural states for the purposes of conserving/regulating ecosystem services and expanding the spatial extent of natural landscapes that have been lost due to previous human activity. Includes actions to create new ecosystems in place of a naturally occurring one or where one does not exist.*  *Examples include reforestation, afforestation, passive restoration, etc...* |

**Table 4.** Complementary intervention typology (see here for [detailed typology](https://docs.google.com/spreadsheets/d/1ASQwVH-AIB7qlXX6DTd7_MsMIndaWLtPoJClhaLmsvM/edit#gid=0))

| **Category** | **Definition** |
| --- | --- |
| Policies, laws, mandates, and regulation | Actions to develop, change, influence, and help implement formal legislation, regulations, and voluntary standards aimed at supporting climate change mitigation actions |
| Training, technical support, and capacity building | Actions to build capacity to do better conservation including developing partnerships and institutions as well as improving understanding and skills, and influencing behavior |
| Good governance and securing rights | Actions taken to define and secure rights to resources by local actors, build local capacity for management and participation and empowerment in decision-making, improving and strengthening governance structures and processes to ensure fair and equitable participation, inclusion, transparency, and accountability in management of natural resources and ecosystems |
| Livelihood, economic & other incentives | Actions to use economic and other incentives to influence behaviour around climate change mitigation actions |

**Table 5 Socio-economic outcomes (including provisioning and cultural ecosystem services)**

| **Category** | **Definition** |
| --- | --- |
| *Economic well-being* | This encompasses the living standards of basic life including both economic and material necessities.  Economic living standards: Income, employment, employment opportunities, wealth, poverty, savings  Material living standards: Material assets owned, basic infrastructure (electricity, water, telecommunication and transportation), shelter, resource use (this can include sustainable use/harvest) |
| *Health* | Any component of individual mental or physical health or access to health services |
| *Safety and security* | Covers any component of physical security (threat to personal body and community sense of safety), and resilience and/or adaptive capacity to respond to changing environments and shocks |
| *Rights and empowerment* | Structures and processes for decision-making that include both formal and informal rules and ability of individuals and groups to be heard and participate in formal and informal decision-making processes. Includes changes to de jure and de facto bundle of rights to resources and ability to exercise. |
| *Education and skills* | Includes both formal and information education and training outcomes as well as educational infrastructure. Includes changes in awareness of climate change and/or environmental issues |
| *Social capital* | Includes measures of the networks of relationships among individuals and groups that live and work within a particular society and include relations to 'external' groups (such as foreign implementers) as well as the 'state' (formal government at various scales) |
| *Culture* | Cultural, societal and traditional values related to natural resources and nature to an individual, group, and/or community, spiritual and aesthetic values, livelihoods, social relations, sense of place, cultural heritage, etc. |
| *Local and traditional knowledge* | Knowledge, know-how, skills and practices developed and transmitted across generations within a community, frequently contributing to cultural or spiritual identity. |
| *Agricultural productivity* | Agricultural productivity is typically measured in crop yield - the harvested production per unit of harvested area for crop products (ABARES 2021). In this category we also include measures of crop growth and survival. |

**Table 6.** Biological and ecological outcomes typology (see [detailed typology here](https://docs.google.com/spreadsheets/d/1XAvwnwtABM1g06Mj9f0tjMq480PVOtUiCwtpmstica4/edit#gid=1123334850))

| **Category** | **Definition** |
| --- | --- |
| *Population/species* | Outcomes focused on change in populations of individuals or populations within species |
| *Ecological community* | Outcomes focused on change in community conditions |
| *Ecosystem function* | Outcomes focused on change in ecosystem processes and conditions, includes regulating ecosystem services (e.g. mediation of waste, toxins, and other nuisances; mediation of flows; maintenance of physical, chemical, and biological conditions including life cycle, disease, soil formation, water and climate regulation, etc...) |
