# Supplementary material for: What evidence exists on the links between natural climate solutions and climate change mitigation outcomes in subtropical and tropical terrestrial regions? A systematic map protocol
Source: Environ Evid. 2022 Apr 19;11:15. doi: 10.1186/s13750-022-00268-w (PMC9017726; doi:10.1186/s13750-022-00268-w)
Supplement: Supplementary file 4 — Additional file 4: Protocol for backwards citation chasing. [file 13750_2022_268_MOESM4_ESM.docx]

**Natural climate solutions systematic map**

*Review of reviews for backwards citation chasing protocol*

**Overarching Key Question:**

What is the evidence base for the links between natural climate solutions (NCS) interventions and impacts on land and forest management practices, and climate change mitigation outcomes in forest, agricultural, and grassland landscapes in tropical regions?

**Objective of this sub-protocol:**

Often it is useful to begin a synthesis by looking for relevant reviews that overlap with your review question in order to determine what types of literature may already exist in the evidence base and use it as a basis for backwards citation chasing. Citation chasing is a search approach that uses a list of relevant studies as the source within which to search for relevant articles. *Backwards citation chasing* involves looking at the citations that relevant articles cite in their work and screening *those* citations for inclusion in your synthesis. Similarly, *forwards citation chasing* involves looking at who cites the relevant articles you have identified and screening *those* citations for inclusion in your synthesis. Since relevant articles to your synthesis question are likely citing other relevant articles, this is a good way to begin to explore the evidence base.

Starting your search strategy using this approach (that we are calling a “review of reviews for backward citation chasing”) allows you to identify an initial evidence base that is more likely to be relevant. This can help you:

- Refine your search strategy that you will use to find primary articles
- Identify articles for your test library
- Refine the scope of your question/sub-questions and inclusion criteria
- Train machine learning models in your synthesis platform to better identify relevant articles in your main search strategy

This review of reviews has two purposes: (1) identify relevant reviews for the overarching key questions and (2) identify opportunistically reviews that are relevant for the umbrella review stage of the main systematic map project.

**Methods**

***Phase 1: Searching for relevant reviews***

In this sub-project, we will identify potentially relevant reviews from specialist databases, expert solicitation, and a targeted search within a bibliographic database.

Searches for relevant published academic and grey literature will be performed in the English language with predefined search terms. The searches will be conducted in three stages (i) new searches in bibliographic databases and specialist databases, including online publication databases and organisational websites, (ii) screening through a previous collation of syntheses relevant to environment and conservation topics, (iii) expert solicited syntheses.

*(i) Searches in bibliographic databases and specialized databases/Journals*

We will search the following databases for articles:

1. Web of Science Core collections (published in the last 3 years (2018-present))
2. CEE Database of Evidence Reviews
3. Campbell Collaboration
4. 3ie
5. Environmental Evidence

We use the following Boolean search string (English) to search in Web of Science:

TS=(forest OR woodland OR meadow OR pasture OR agricultur* OR rangeland OR grassland OR mangrove OR tree OR cropland OR grazing OR land OR ecosystem OR landscape) AND TS=("manure management" OR "cropland management" OR windbreaks OR "grazing optimization" OR browsing OR fencing OR exclosures OR ((integrated OR sustainable) NEAR/2 management) OR agroforestry OR silvopastor* OR silvopastur* OR agro-ecology OR "conservation agriculture" OR "tree planting" OR "nutrient management" OR "rice cultivation" OR "cattle management" OR "cover crop" OR "green infrastructure" OR "extended harvest cycles") AND TS=("systematic review" OR "systematic map" OR meta-analysis OR meta-analyses OR "evidence review" OR "evidence gap map" OR "systematic literature review" OR "systematic evidence review") NOT TS=("soil")

The remaining sources were searched as following:

| CEEDER | forest OR woodland OR meadow OR pasture OR agriculture OR agricultural OR rangeland OR grassland OR mangrove OR tree OR cropland OR grazing OR land FILTERED BY criteria 3.1 > 1 and criteria 4.3 > 1 |
| --- | --- |
| 3ie | Filter all by: systematic review OR evidence gap map IN environment and natural resource management |
| Campbell | Filter all by: systematic review OR evidence gap map AND English |
| Environmental Evidence Journal | Searched all by eye, screened on title |
| Existing database of reviews (Cons-HWB) | Filter by tag: climate change, bio/eco, hwb, behavior, agriculture, OR land-use |

All searches were run on 06/30/2021.

*(iii) Previous reviews of reviews*

We will search a curated list of existing reviews in environmental and conservation science compiled using a systematic search strategy in 2020.

In brief, these syntheses were compiled using the following search strategy run in English:

**Search terms:**

("nature" OR "environment" OR "environmental" OR "conservation" OR "environmental management" OR "water security" OR "agriculture" OR "forestry" OR "fisheries" OR "natural resources" OR "natural resource management" OR "biodiversity" OR "climate change" OR "ecosystem" OR "ecosystem services" OR "pollution" OR "invasive species" OR "endangered species" OR "sustainability" OR "soil management" OR "watershed" OR "environmental legislation" OR "environmental education" OR "environmental justice" OR "community-based" OR "agroforestry" OR "forest" OR "marine" OR "freshwater" OR "desert" OR "grassland" OR "coastal" OR "coral reef" OR "mangrove" OR "seagrass" OR "beach")

AND

("systematic map" OR "systematic review" OR "evidence map" OR "evidence gap map" OR "rapid review" OR "rapid evidence synthesis")

**Databases:**

The following databases and websites were searched using the above search string where applicable. Websites with limited Boolean search capacity were searched using a few terms at a time and/or using relevant filters for study type and topic area (e.g. 3ie and Campbell). All searches were run in April 2020.

- Journal of Environmental Evidence
- CEE Database of Evidence Reviews (CEEDER)
- 3ie Evidence Portal
- Campbell registered and completed syntheses
- Environment Complete (database)
- Web of Science (database)

The reviews in this list were screened at title and abstract and tagged based on the description of their topic areas. We will search a subset of these reviews that include any of the following tags:

| *hwb* | Impacts of intervention/exposure on human well-being (e.g. social, economic, cultural/spiritual, governance, health, security) |
| --- | --- |
| *bio/eco* | Impacts of intervention/exposure on biological/ecological impacts |
| *behavior* | Impacts of intervention/exposure on human behavior (e.g. compliance, awareness, willingness to participate/pay, adoption of practices, etc…) |
| *agriculture* | Impacts of agriculture/food systems, agricultural management |
| *land-use* | Impacts of land-use regimes and land-use management (e.g. dams, environmental flows) |
| *climate change* | Impacts of climate change/climate change mitigation/management |

(iii) Expert solicitation

Through our *stakeholder advisory group* and the *review team, we compiled a list of* potentially relevant reviews.

Following completion of the search strategy, a total of 913 search results were downloaded. Following deduplication, 709 unique citations were included for screening.

**Phase 2: Defining the Inclusion/exclusion criteria**

*Inclusion criteria*

To be included in the review of reviews, studies must meet the criteria outlined below.

**Table 1.** Working inclusion and exclusion criteria

|  | **Included** | **Excluded** |
| --- | --- | --- |
| **Population** | Terrestrial ecosystems in tropical countries | Marine, freshwater (rivers, streams, riparian, floodplains), coastal ecosystems (except for mangroves)  Reviews on exclusively non-tropical countries  Urban and peri-urban settings  Verges/roadside/hedgerows |
| **Intervention** | Land stewardship interventions that aim to protect, manage, or restore existing natural terrestrial ecosystems  Land stewardship interventions that aim to create or manage new ecosystems (e.g. afforestation, plantation forests, replanting with non-native plants, constructed ecosystems, artificial grasslands, natural/green infrastructure) in non-urban/peri-urban areas. This can include land abandonment for passive regeneration.  Interventions that aim to promote and implement sustainable and/or climate-smart agriculture, grazing, and agroforestry management and practices  Land tenure may be considered if it is within the context of one of the above interventions  Incentives (e.g. economic, material, governance, cultural, etc…) may be considered if it is explicitly examined within the context of one of the above interventions | Contribution of existing ecosystems (without an intervention)  Hybrid natural/engineered interventions  Effectiveness of complementary interventions (e.g. training, capacity building, governance, equity, incentives, policies, monitoring and enforcement, payments for ecosystem services) without explicit tie to land stewardship intervention  Interventions that focus on belowground biomass (e.g. soil, watershed, submerged vegetation)  Climate change policies and regulations  Supply chain activities  Activities focused on climate change adaptation  General agricultural activities including conventional intensification, buffer and strip management, etc...  Bioenergy, alternative fuels, etc.. that aim to address climate change but are not nature-based interventions. This includes alternative cookstoves and biogas interventions |
| **Study type + comparator** | Systematic maps and reviews, evidence gap maps, or any literature review that clearly documents their search strategy and provides a list of included articles | Primary studies, modeling studies, opinions, editorials, non-systematic reviews or maps, or any review that does not document their search strategy and does not provide a list of included articles  Protocols for reviews |
| **Outcome** | Environmental outcomes directly related to climate change mitigation (GHG emissions and carbon storage and sequestration above ground)  Land and forest management practice outcomes | Studies that address climate change impact on human health with no NCS intervention  Studies that only examine changes to one of four outcomes WITHOUT looking at an NCS intervention  **Regulating and supporting ecosystem services** including nutrient cycling, soil formation, primary productivity, climate, flood, temperature regulation, fire regulation, water quality and flows  **Provisioning and cultural ecosystem service** outcomes including crop yield, fodder, productivity, pest control, etc… and aesthetic, recreational, educational, spiritual values, etc…with no NCS intervention  Climatic outcomes (e.g. precipitation, temperature, etc…)  Physical outcomes (e.g. evapotranspiration, water filtration, etc…)  Socio-economic outcomes  Biological and ecological outcome |

**Phase 3: Screening reviews at title and abstract**

Screening at title and abstract occurred in colandr (Cheng et al. 2018) across a team of 2 reviewers (SC, ES). The team conducted a set of side-by-side training sessions to ensure consistency on eligibility criteria.

Following full text screening, a total of 115 potentially relevant reviews were included.

**Phase 4: Screening at full text**

Full text inclusion criteria are the same as inclusion criteria at title and abstract, however, we will be specifically assessing whether the reviews:

- Include if it contains list of search terms AND where they searched
- Include if it contains list of included articles (beyond listing them within the references)

These criteria are based on the Amber criteria of the Collaboration for Environmental Evidence Systematic Appraisal Tool (CEESAT) (Konno et al. 2020).

Following full text screening, a total of 39 relevant syntheses were included (see below) and their included articles (n=4,045 citations) were added to the list of search results to be screened for the primary systematic map (backwards citation screening).

**Included relevant syntheses for backwards citation chasing**

| A global meta-analysis on the ecological drivers of forest restoration success | 2016 | Crouzeilles et al. 2016 |
| --- | --- | --- |
| Achieving the SDGs in Africa: A Cross-sectoral Evidence Gap Map | 2020 | Doherty et al. 2020 |
| Biotechnologies in agriculture and forestry: Governance insights from a comparative systematic review of barriers and recommendations | 2020 | Pelai et al. 2020 |
| Can agroforestry systems enhance biodiversity and ecosystem service provision in agricultural landscapes? A meta-analysis for the Brazilian Atlantic Forest | 2019 | Santos et al. 2019 |
| Carbon accumulation in agroforestry systems is affected by tree species diversity, age and regional climate: A global meta-analysis | 2020 | Ma et al. 2020 |
| China's conversion of cropland to forest program: a systematic review of the environmental and socioeconomic effects | 2016 | Gutierrez Rodriguez et al. 2016 |
| Co-benefits of greenhouse gas mitigation: A review and classification by type, mitigation sector, and geography | 2017 | Deng et al. 2017 |
| Community managed forests and forest protected areas: An assessment of their conservation effectiveness across the tropics | 2012 | Porter-Bolland et al. 2012 |
| Cover Cropping: A Malleable Solution for Sustainable Agriculture? Meta-Analysis of Ecosystem Service Frameworks in Perennial Systems | 2021 | Creze and Horwath 2021 |
| Do timber plantations contribute to forest conservation? | 2016 | Pirard et al. 2016 |
| Does the gender composition of forest and fishery management groups affect resource governance and conservation outcomes? A systematic map | 2016 | Leisher et al. 2016 |
| Drivers of tropical forest cover increase: A systematic review | 2020 | Borda-Niño et al. 2020 |
| Ecological restoration success is higher for natural regeneration than for active restoration in tropical forests | 2017 | Crouzeilles et al. 2017 |
| Effectiveness of terrestrial protected areas in reducing habitat loss and population declines | 2013 | Geldmann et al. 2013 |
| Effects of decentralized forest management (DFM) on deforestation and poverty in low- and middle-income countries: a systematic review | 2014 | Samii et al. 2014 |
| Emerging Evidence on the Effectiveness of Tropical Forest Conservation | 2016 | Börner et al. 2016 |
| Evaluating land use and livelihood impacts of early forest carbon projects: Lessons for learning about REDD+ | 2011 | Caplow et al. 2011 |
| Examining the evidence base for forest conservation interventions | 2016 | Puri et al. 2016 |
| Gender and conservation agriculture in sub-Saharan Africa: a systematic review | 2019 | Wekesah et al. 2019 |
| How have carbon stocks in central and southern Africa’s miombo woodlands changed over the last 50+ years? A systematic map of the evidence | 2018 | Gumbo et al. 2018 |
| Impact of agronomy practices on the effects of reduced tillage systems on CH4 and N2O emissions from agricultural fields: A global meta-analysis | 2018 | Feng et al. 2018 |
| Incentives for climate mitigation in the land use sector: the effects of payment for environmental services on environmental and socioeconomic outcomes in low- and middle-income countries: A mixed-methods systematic review | 2019 | Snilsveit et al. 2019 |
| Irrigation and greenhouse gas emissions: A review of field-based studies | 2020 | Sapkota et al. 2020 |
| Land-use change and forestry programmes: evidence on the effects on greenhouse gas emissions and food security | 2016 | Snilsveit et al. 2016 |
| Mapping the effectiveness of nature-based solutions for climate change adaptation | 2020 | Chausson et al. 2020 |
| Measuring impact of protected area management interventions: current and future use of the Global Database of Protected Area Management Effectiveness | 2015 | Coad et al. 2015 |
| Meta-analysis of yield and nitrous oxide outcomes for nitrogen management in agriculture | 2021 | Scasta et al. 2021 |
| Mitigation of carbon and nitrogen losses during pig manure composting: A meta-analysis | 2021 | Zhang et al. 2021 |
| Reconsidering the efficiency of grazing exclusion using fences on the Tibetan Plateau | 2020 | Sun et al. 2020 |
| Restoration of ecosystem services in tropical forests: A global meta-analysis | 2018 | Shimamoto et al. 2018 |
| The Effectiveness of Forest Conservation Policies and Programs | 2020 | Börner et al. 2020 |
| The environmental, socioeconomic, and health impacts of woodfuel value chains in Sub-Saharan Africa: a systematic map | 2017 | Sola et al. 2017 |
| The evidence base for Community Forest Management as a mechanism for supplying global environmental benefits and improving local welfare | 2010 | Bowler et al. 2010 |
| The impacts of agroforestry on agricultural productivity, ecosystem services, and human well-being in low-and middle-income countries: An evidence and gap map | 2020 | Miller et al. 2020 |
| The place of community forest management in the REDD+ landscape | 2016 | Pelletier et al. 2016 |
| What Drives Deforestation and What Stops It? A Meta-Analysis | 2017 | Busch et al. 2017 |
| What evidence exists for the effectiveness of on-farm conservation land management strategies for preserving ecosystem services in developing countries? A systematic map | 2016 | Thorn et al. 2016 |
| What evidence exists on the impact of governance type on the conservation effectiveness of forest protected areas? Knowledge base and evidence gaps | 2015 | Macura et al. 2015 |
| What works in tropical forest conservation, and what does not: Effectiveness of four strategies in terms of environmental, social, and economic outcomes | 2019 | Burivalova et al. 2019 |
